# Association of Preoperative Clinical, Laboratory, Imaging, and Pathologic Data With Clinically Beneficial Pathology Among Routine Splenectomy Specimens

**DOI:** 10.1001/jamanetworkopen.2021.20946

**Published:** 2021-08-16

**Authors:** Lee Erez, Ginette Schiby, Imri Amiel, Shachar Naor, Naama Keren, Danny Rosin, Iris Barshack, Jonathan Canaani

**Affiliations:** 1 Hematology Division, Chaim Sheba Medical Center, Tel-Hashomer, Faculty of Medicine, Tel Aviv University, Tel Aviv, Israel; 2 Department of Pathology, Chaim Sheba Medical Center, Tel-Hashomer, Faculty of Medicine, Tel Aviv University, Tel Aviv, Israel; 3 Department of Surgery and Transplantation, Chaim Sheba Medical Center, Tel-Hashomer, Faculty of Medicine, Tel Aviv University, Tel Aviv, Israel

## Abstract

**Importance:**

Previous studies have shown that uniform pathologic review of all splenectomy surgical specimens reveals new clinically actionable diagnoses only in a minority of cases.

**Objective:**

To examine whether the aggregate of clinical, laboratory, imaging, and pathologic preoperative data is associated with a clinically beneficial pathologic study for routine splenectomy surgical specimens.

**Design, Setting, and Participants:**

This single-center retrospective cohort study included all patients who underwent splenectomy from January 1, 2013, through December 31, 2018, at a single center. Clinical, imaging, and pathologic data were extracted from the institution’s electronic medical records system. Data analysis was conducted from June to November 2020.

**Exposures:**

Undergoing splenectomy for trauma or diagnostic or therapeutic indications.

**Main Outcomes and Measures:**

Spleen pathology study resulting in a new medical diagnosis or change in medical management.

**Results:**

Overall, 90 patients (53 [59%] men) with a median (range) age of 59 (19-90) years underwent splenectomy for therapeutic purposes in 41 patients (45%), trauma in 24 patients (27%), diagnostic purposes in 15 patients (17%), and combined therapeutic and diagnostic purposes in 9 patients (10%). In 14 patients (15%) a new malignant neoplasm was found, and in 8 patients (9%), a new nonneoplastic medical condition was diagnosed. A new pathologic diagnosis resulted in change in medical management in 16 patients (18%). In patients without a prior diagnosis of cancer, 41 of 56 pathology biopsies (73%) were found to be normal whereas in 7 biopsies (13%), a new diagnosis of a hematologic malignant neoplasm was revealed (*P* < .001). Patients with clinical splenomegaly were significantly more likely to have a new pathologic diagnosis of cancer compared with patients without splenomegaly (15 of 26 [58%] vs 4 of 64 [7%]; *P* < .001). In 39 of 43 patients (91%) with normal presurgery imaging studies, normal spleen pathology was revealed, whereas in 14 of 17 patients (82%) with abnormal imaging studies, a new hematological malignant neoplasm was diagnosed following pathologic review of the spleen specimen (*P* < .001). Patients with gross abnormalities on macroscopic examination had a significantly increased likelihood of a hematological cancer diagnosis (17 of 40 [43%]) and a solid cancer diagnosis (4 [10%]) compared with patients with grossly normal specimens (4 of 49 [8%]; *P* < .001).

**Conclusions and Relevance:**

In this cohort study, routine pathologic review of spleen specimens was clinically beneficial in patients with splenomegaly, abnormal imaging results, a prior diagnosis of cancer, and with grossly abnormal spleens.

## Key Points

**Question:**

What are the clinical, laboratory, imaging, and gross pathologic data associated with a high likelihood of a clinically beneficial pathologic study for routine splenectomy surgical specimens?

**Findings:**

In this cohort study of 90 patients who underwent splenectomy for diagnostic, therapeutic, or trauma-related reasons, patients presenting with clinical splenomegaly, abnormal presurgery imaging results, a prior diagnosis of cancer, or grossly abnormal spleens were significantly more likely to have a new cancer diagnosis revealed or incur a change in medical management as a result of the spleen pathology study.

**Meaning:**

These findings suggest that routine pathologic review of splenectomy specimens is mostly warranted for patients with known clinical splenomegaly, abnormal presurgery imaging results, a prior diagnosis of cancer, or with grossly abnormal spleens.

## Teaser

This cohort study examines whether the aggregate of clinical, laboratory, imaging, and pathologic preoperative data is associated with a clinically beneficial pathologic study for routine splenectomy surgical specimens.

## Introduction

A nearly universal dictum of medical care is that all specimens removed during surgery are routinely sent to pathological review with the intent of finding a new previously undiagnosed medical condition. As the spleen is the most frequently injured organ after blunt trauma,^1^ spleen surgical specimens are commonly sent for pathology review even in the absence of clinical or imaging data suggesting a specific disease involving the spleen. Several retrospective studies have addressed the clinical yield of this approach, indicating that only 0.5% to 1.2% of patients who underwent splenectomy for trauma-related reasons had new clinically significant findings found on pathology review.^2,3,4,5^ Conversely, in patients undergoing diagnostic splenectomy, typically for the workup of splenomegaly or discrete mass lesions of the spleen, much higher rates of a new pathologic diagnosis are established, with lymphoma being a predominant etiology.^2,6,7,8,9^ Owing to these data, the practice of routine uniform pathology review of all surgical spleen specimens has been questioned. Previous publications have identified several features suggestive of a yet-undiagnosed medical condition, such as spleen size,^9^ macroscopic appearance,^3,4^ and patient age^5^; however, to our knowledge no comprehensive assessment of all clinical factors associated with a diagnostic spleen pathology review has yet been performed. Hence, the specific patient segment that would benefit the most from routine pathology review of splenectomy specimens has not been precisely defined. In this analysis of 90 consecutive splenectomies, we sought to define clinical, laboratory, and imaging results associated with the diagnosis of a new medical condition or with a change in medical management.

## Methods

### Study Design

This was a retrospective cohort study of 90 consecutive adult patients who underwent splenectomy from January 1, 2013, through December 31, 2018, at the Chaim Sheba Medical Center. Using our center’s electronic medical record systems, patient medical records were reviewed, and demographic, clinical, and laboratory data were collected. This study was approved by the hospital’s institutional review board, which granted a waiver of informed consent because the data were deidentified and the study posted minimal risk to participants. The study was conducted in accordance with the Strengthening the Reporting of Observational Studies in Epidemiology (STROBE) reporting guidelines.^10^

### Patients and Procedure

Our primary exposure was patients undergoing splenectomy for trauma or diagnostic or therapeutic indications at our medical center, with the main outcomes and measures being spleen pathology studies resulting in a new medical diagnosis or a change in medical management. All specimens were evaluated in the pathology department and underwent gross and histological examination. All specimens were weighed and measured in 3 dimensions. Gross examination included description and documentation of defects or pathology. Indications for splenectomy were categorized as trauma related, diagnostic, therapeutic, or as combined intent for diagnostic and therapeutic purposes. Baseline laboratory values were those obtained at the day of splenectomy and included white blood cell count (reported as cells per microliter [to convert to ×10^9^ per liter, multiply by 0.001]), hemoglobin level (reported as grams per deciliter [to convert to grams per liter, multiply by 10.0]), and platelet count (reported as ×10^9^ per microliter [to convert to ×10^3^ per liter, multiply by 1.0]). Presurgery imaging results were reviewed by the leading author (L.E.) and, after careful review of the clinical medical record, were assessed for clinical relevance to the indication for splenectomy. A new treatment decision was defined as a change in medical management based on findings in a pathology review of the excised spleen. Surgical outcomes assessed were duration of hospital admission, surgical complications, and the 30-day mortality rate following splenectomy. Surgical complications were classified according to societal guidelines.^11^

### Statistical Analysis

Comparison between categorical variables between groups was performed using Fisher exact test and Pearson χ^2^ test. Continuous variables were compared with 1-way analysis of variance with post hoc analysis with Tukey *b* test. All tests were 2-sided with the type I error rate set at .05. Statistical analyses were performed using the SPSS statistical software version 25.0 (IBM Corp).

## Results

### Baseline Patient and Surgical Data

Characteristics of the 90 patients who underwent splenectomy in our center are summarized in Table 1. The median (range) patient age was 59 (19-90) years, and 53 patients (59%) were men. Thirty-four patients (38%) had a prior diagnosis of malignant neoplasm, with lymphoma and solid cancers accounting for most diagnoses (16 and 15 patients, respectively). The indications for splenectomy were therapeutic in 41 patients (45%), trauma related in 24 patients (27%), diagnostic in 15 patients (17%), and combined therapeutic and diagnostic in 9 patients (10%). In nearly half of the analyzed cohort (43 [48%]), presurgery imaging results were contributary to the decision to pursue splenectomy, whereas in 30 patients, a splenic mass (13 [14%]) or splenomegaly (17 [19%]) was detected in imaging studies. As outlined in Table 2, most patients (66 [73%]) underwent elective surgery with 39 (43%) having laparoscopic splenectomy; 41 (46%), open splenectomy; and 10 (11%), conversion of laparoscopy to open splenectomy. The median (range) duration of hospital admission was 8 (3-120) days, with a 30-day mortality rate of 2% (2 patients). Notably, mean (SD) preoperative hemoglobin levels were significantly lower in patients who died during their hospital admission compared with those who did not (9.0 [0.7] g/dL vs 12.0 [1.9] g/dL; *P* = .03). Patients undergoing elective splenectomy were more likely to have lower mean (SD) white blood cell and platelet counts compared with patients who had emergency splenectomy performed (white blood cell count: 9800/μL [6370] vs 15 500/μL [6510]; *P* < .001; platelet count: 173 [103] ×10^3^/μL vs 213 [68] ×10^3^/μL; *P* = .04). Additionally, mean (SD) spleen weight as well as mean (SD) spleen volume were significantly increased in the elective surgery setting compared with emergency surgery (weight: 781 [981] g vs 194 [118] g; *P* < .001; volume: 1673 [2077] cm^3^ vs 416 [253] cm^3^; *P* < .001). Patients who underwent emergency splenectomy were younger than those who underwent elective splenectomy, but the difference was not statistically significant (46 [25] years vs 54 [17] years; *P* = .08) (eTable 1 in the Supplement). Furthermore, clinically noted splenomegaly were uniformly operated in the elective setting (17 [100%]) compared with patients without clinical splenomegaly, in whom emergency surgery was performed in 24 of 63 patients (38%) (*P* < .001). As shown in eTable 2 in the Supplement, patient age, hemoglobin level, spleen size, and spleen volume were not significantly different between patients who underwent a laparoscopic splenectomy compared with those who had open surgery performed. However, patients who underwent an open procedure had a significantly higher mean (SD) WBC count (13 600/μL [7140] vs 9400/μL [4890]; *P* = .02) and a higher mean (SD) platelet count (211 [91] ×10^3^/μL vs 157 [84] ×10^3^/μL; *P* = .03) compared with patients who underwent laparoscopic splenectomy.

**Table 1.** Baseline Characteristics

Clinical parameter	Patients, No. (%) (N = 90)
Age, median (range), y	59 (19-90)
Gender	
Male	53 (59)
Female	37 (41)
Medical background	
Lymphoma	16 (18)
ITP	9 (10)
Other	46 (51)
Solid malignant neoplasm	15 (17)
Autoimmune disorder	4 (4)
Alive at the time of analysis	74 (82)
Baseline laboratory parameters, median (range)	
Hemoglobin, g/dL	12 (6.7-15.6)
WBC, /μL	10 200 (1500-36 700)
Platelets, ×10^3^/μL	173 (2-413)
Prior malignant neoplasm	34 (38)
Clinical splenomegaly	26 (29)
Indication for splenectomy	
Trauma	24 (27)
Diagnostic	15 (17)
Therapeutic	41 (45)
Other	1 (1)
Diagnostic and therapeutic	9 (10)
Presurgery imaging results	
Not relevant	43 (48)
Splenic mass	13 (14)
Splenomegaly	17 (19)
Pancreatic cyst or mass	11 (12)
Other tumor	6 (7)

Abbreviations: ITP, immune thrombocytopenic purpura; WBC, white blood cells.

SI conversion factors: To convert hemoglobin to grams per liter, multiply by 10.0; platelets to ×10^9^ per liter, multiply by 1.0; WBC to ×10^9^ per liter, multiply by 0.001.

**Table 2.** Surgical Data

Clinical parameter	Patients, No. (%) (N = 90)
Timing of surgery	
Elective	66 (73)
Emergency	24 (27)
Surgery type	
Laparoscopy	39 (43)
Laparotomy	41 (46)
Converted	10 (11)
Duration of hospital admission, median (range), d	8 (3-120)
Surgical complications	27 (30)
30-d mortality rate	2 (2)

### Pathology Findings

In 40 patients (45%), a macroscopic abnormality was noted in initial pathologic inspection (Table 3). In most patients (68 [76%]), no new medical diagnosis was detected following histologic analysis; however in 14 patients (15%), a new malignant neoplasm was found, and in 8 patients (9%) a new nonneoplastic medical condition was diagnosed. A new pathologic diagnosis resulted in change in medical management in 16 patients (18%). In patients without a prior diagnosis of cancer, 41 of 56 pathology biopsies (73%) were found to be normal, whereas in 7 biopsies (13%), a new diagnosis of a hematologic malignant neoplasm was revealed (*P* < .001). Clinically detectable splenomegaly prior to splenectomy was significantly associated with a new pathologic diagnosis of a hematologic cancer in contrast to patients without clinical splenomegaly, for whom only a minority of patients had diagnoses with a new hematologic cancer (15 of 26 [58%] vs 4 of 64 [7%]; *P* < .001). Imaging performed prior to surgery was a useful adjunct in estimating the likelihood of a new pathologic diagnosis, as 39 of 43 patients (91%) with normal imaging studies were found to have a normal spleen pathology study, whereas 14 of 17 patients (82%) with splenomegaly on imaging were found to have a hematological malignant neoplasm (*P* < .001). Patients with spleens detected with gross abnormalities on macroscopic examination had a significantly increased likelihood of a hematological cancer diagnosis (17 of 40 [43%]) and a solid cancer (4 [10%]) compared with grossly normal specimens (4 of 49 [8%]; *P* < .001). As outlined in Table 4, patients with a hematologic cancer diagnosis were more likely to have increased mean (SD) spleen weights compared with patients with a diagnosis of a solid malignancy or normal pathology spleens (1527 [1335] g vs 293 [152] g and 276 [271] g, respectively; *P* < .001). Patients with hematologic cancers were also seen to have significantly lower blood counts compared with patients with solid malignant neoplasms and normal pathology spleens, whereas patient age was not significantly different between groups.

**Table 3.** Pathology Data

Clinical Parameter	Patients, No. (%) (N = 90)
Spleen, median (range)	
Weight, g	300 (60-5396)
Volume, cm^3^	668 (77-11 088)
Macropathological irregularity	40 (45)
Spleen pathology resulting in new diagnosis	
New neoplasia diagnosed	14 (15)
New nonneoplastic diagnosis	8 (9)
No new diagnosis	68 (76)
Spleen pathology diagnosis resulted in new management decision	16 (18)

**Table 4.** Analysis of Variance Comparison of Clinical Parameters According to Pathology Result

Variable	Mean (SD)				*F*	*P* value
	Normal pathology	Hematologic malignancy	Solid malignancy	Other		
Age, y	49.74 (22.3)	59 (12.6)	55 (14.6)	49.26 (16.3)	1.19	.32
WBC, /μL	13.48 (6.6)	5.99 (3.9)	13.58 (13.4)	9.87 (3.6)	6.73	<.001
Hemoglobin, g/dL	12.02 (1.9)	11.09 (1.8)	13.99 (1.2)	12.31 (1.4)	3.65	.02
Platelets, ×10^3^/μL	190.2 (90.6)	128.2 (88.0)	224.8 (70.5)	209.4 (106.7)	3.09	.03
Spleen						
Weight, g	276.26 (271.2)	1527.94 (1335.0)	293.20 (152.3)	464.90 (322.1)	14.387	<.001
Volume, cm^3^	553.24 (469.1)	3365.56 (2893.6)	1434.00 (1444.1)	878.13 (731.3)	14.115	<.001

Abbreviation: WBC, white blood cells.

SI conversion factors: To convert hemoglobin to grams per liter, multiply by 10.0; platelets to ×10^9^ per liter, multiply by 1.0; WBC to ×10^9^ per liter, multiply by 0.001.

### Analysis of Factors Associated With the Likelihood of a Clinically Useful Pathological Diagnosis

We then performed a 1-way analysis of variance analysis to assess whether baseline clinical and laboratory data would be significantly different between patients whose splenectomy pathological study revealed a new medical diagnosis compared with those whose pathology results did not result in a new diagnosis. Notably, mean (SD) spleen weight and volume were significantly increased in patients with a new diagnosis of cancer per spleen histology compared with patients with a new nonneoplastic diagnosis or those without a new diagnosis (weight: 1368 [1065] g vs 414 [268] g and 449 [770] g, respectively; *P* = .001; volume: 3082 [2315] cm^3^ vs 820 [531] cm^3^ and 948 [1580] cm^3^, respectively; *P* = .001). Age, white blood cell count, platelet count, and hemoglobin levels did not differ to a significant degree between groups. In terms of a new clinical management decision, only age was significantly different between groups to the extent that patients with a new management decision were significant older than those without a new management decision (mean [SD] age, 59 [14] years vs 50 [20] years; *P* = .03). White blood cell count, platelet count, hemoglobin levels, spleen weight, and spleen volume were not significantly different between groups (Table 5). The likelihood of a new management decision based on splenectomy pathology results in patients with no history of cancer was quite low, seen in only 3 of 55 patients (6%), while in patients with a prior diagnosis of malignant neoplasms, the new pathology resulted in a new management decision in 13 of 32 (41%) (*P* < .001). Reviewing specifically the subset of patients who underwent splenectomy for trauma-related reasons (eTable 3 and eTable 4 in the Supplement), none of the patients had a prior history of malignancy, and only 2 patients had prior relevant medical diagnoses (1 with immune thrombocytopenic purpura and 1 with an autoimmune disorder). None of the patients with trauma were diagnosed with a new medical condition following pathology review of the spleen specimen, and none incurred a new clinical management decision based on the spleen pathology result.

**Table 5.** Analysis of Factors Associated With a New Management Decision

Clinical parameter	Mean (SD)		*P* value
	No new treatment decision	New treatment decision	
Age, y	49.9 (20.4)	59.4 (13.5)	.03
WBC, ×10^3^/μL	11.23 (6.0)	12.65 (9.7)	.46
Hemoglobin, g/dL	12.0 (1.8)	11.7 (2.0)	.47
Platelets, ×10^3^/μL	178.4 (93.2)	219.1 (110.3)	.13
Spleen			
Weight, g	559.8 (715.4)	383.5 (313.8)	.41
Volume, cm^3^	1050.9 (1186.9)	1088.2 (1213.5)	.92

Abbreviation: WBC, white blood cells.

SI conversion factors: To convert hemoglobin to grams per liter, multiply by 10.0; platelets to ×10^9^ per liter, multiply by 1.0; WBC to ×10^9^ per liter, multiply by 0.001.

## Discussion

Concurrent with contemporary guidelines and standard clinical practice,^12^ all surgical specimens of the spleen removed during surgery are uniformly sent for pathology review irrespective of the clinical setting and indication for splenectomy. However, an emerging body of literature indicates that in designated clinical scenarios, namely trauma-related splenectomy, the diagnostic yield and therapeutic gain in terms of change in medical care are for the most part minor. In this analysis, we found that a new diagnosis of malignant neoplasm was detected on pathology review in less than 20% of patients; furthermore, we established specific clinical, pathologic, and imaging parameters associated with an increased likelihood of a new cancer diagnosis and of new clinical management decisions based on the pathology findings.

The role of routine histologic evaluation of splenectomy specimens remains a matter of debate most prominently in the clinical setting of trauma, where previous studies indicate a low probability of detecting previously undiagnosed medical conditions. Indeed, a study by Gertz and colleagues,^5^ using the trauma registry of the Los Angeles County and University of Southern California Medical Center, reviewed 1686 surgical specimens (of which 475 were spleen specimens) obtained during trauma surgeries, and only 0.5% showed a new diagnosis of malignant neoplasm. Importantly, these new pathologic data did not result in changed medical management for any patients, leading the authors to conclude that routine pathology review of specimens obtained during trauma operations is not indicated. In the same vein, previous publications by Laituri et al^4^ and Fakhre et al^3^ show that at least in the setting of trauma-associated splenectomy, all grossly normal appearing spleen specimens are histologically normal. These data are consistent with the findings of this analysis, in which 92% of patients with a normal macroscopic examination did not have abnormal findings on pathology review. Conversely, we found that in patients with clinical splenomegaly and splenomegaly detected on imaging studies, there was a very high likelihood of a new cancer diagnosis (58% and 82%, respectively). In accordance with previous data, we found that patients with hematologic malignant neoplasms had increased spleen weight.^9^

Our cohort consisted of significant patient segment undergoing splenectomy for diagnostic purposes (27%), and thus, it may not be surprising that in nearly one-quarter of our patients, a new diagnosis was established following pathologic review, with 15% of patients being diagnosed with a new malignant neoplasm. In terms of new clinical management decisions based on the pathology findings, we found that older patients were more likely to have new management decisions based on the pathology study. Furthermore, almost all patients (95%) with no prior history of malignant neoplasm did not incur a new management decision following the results of the spleen pathology. In contrast, in patients with a prior diagnosis of malignancy, 40% had a new management decision based on their spleen pathology results.

### Limitations

This study has several limitations. This being a single-center study may have resulted in inadvertent biases inherent to the retrospective nature of the analysis. Additionally, our study cohort consisted of patients with several different indications for splenectomy, ranging from posttrauma patients to patients with a known diagnosis of cancer as well as those who underwent splenectomy as a diagnostic procedure, thus limiting to some degree the ability to generalize our findings to all splenectomy procedures.

## Conclusions

The results of this analysis suggest that preoperative consideration of the aggregate clinical and imaging data, complemented with macroscopic evaluation of spleen specimens, has the potential for faithful indication of the pathologic yield of splenectomy specimens. While previous studies have specifically addressed the yield of spleen specimens in the trauma setting or alternatively in the workup of splenomegaly, we aimed to associate a comprehensively annotated data set consisting of baseline clinical, laboratory, imaging, and pathologic data with the pathologic diagnoses attained with uniform pathologic review of all splenectomy specimens irrespective of the clinical scenario. Our analysis found that younger patients with normal spleens on clinical examination and imaging without a history of cancer and undergoing trauma-related surgery are less likely to be diagnosed with a new medical condition requiring a change in medical management.

## Notes

Supplement. **eTable 1.** Comparison of Clinical Parameters in Patients Undergoing Elective Splenectomy vs Emergency Surgery **eTable 2. **ANOVA Test Comparisons Among Patients According to Surgery Type **eTable 3. **Baseline Characteristics of Trauma Patients Who Underwent Splenectomy **eTable 4. **Surgical-Pathology Data for Trauma Patients Who Underwent Splenectomy
