# Supplementary material for: Association of Preoperative Clinical, Laboratory, Imaging, and Pathologic Data With Clinically Beneficial Pathology Among Routine Splenectomy Specimens
Source: JAMA Netw Open. 2021 Aug 16;4(8):e2120946. doi: 10.1001/jamanetworkopen.2021.20946 (PMC8369355; doi:10.1001/jamanetworkopen.2021.20946)
Supplement: Supplement. — eTable 1. Comparison of Clinical Parameters in Patients Undergoing Elective Splenectomy vs Emergency Surgery eTable 2. ANOVA Test Comparisons Among Patients According to Surgery Type eTable 3. Baseline Characteristics of Trauma Patients Who Underwent Splenectomy eTable 4. Surgical-Pathology Data for Trauma Patients Who Underwent Splenectomy [file jamanetwopen-e2120946-s001.pdf]

Supplemental Online Content

Erez L, Schiby G, Amiel I, et al. Association of preoperative clinical, laboratory, imaging, and pathologic data with clinically beneficial pathology among routine splenectomy specimens. *JAMA Netw Open.* 2021;4(8):e2120946. doi:10.1001/jamanetworkopen.2021.20946

eTable 1. Comparison of Clinical Parameters in Patients Undergoing Elective Splenectomy vs Emergency Surgery

eTable 2. ANOVA Test Comparisons Among Patients According to Surgery Type

eTable 3. Baseline Characteristics of Trauma Patients Who Underwent Splenectomy

eTable 4. Surgical-Pathology Data for Trauma Patients Who Underwent Splenectomy

This supplemental material has been provided by the authors to give readers additional information about their work.

© 2021 Erez L et al. *JAMA Network Open.*

eTable 1. Comparison of Clinical Parameters in Patients Undergoing Elective Splenectomy vs Emergency Surgery

Clinical Parameter	Age	WBC (X 10^3/µL)	Hemoglobin (g/dL)	Platelets (X 10^3/µL)	Spleen weight (gm)	Spleen volume (cm^3)
Elective surgery	54.4	9.8	11.9	173	781.0	1673.1
Emergency surgery	46.3	15.5	12.1	213	193.6	416.2
P	0.081	0.0004	0.65	0.04	0.0001	0.005

WBC, white blood cells

© 2021 Erez L et al. *JAMA Network Open.*

eTable 2. ANOVA Test Comparisons Among Patients According to Surgery Type

Variable	Laparoscopy	Laparotomy	Converted surgery	F	*P*
Age	52.1	50.1	61.2	1.30	0.28
WBC (X 10^3/μL)	9.4	13.6	9.5	4.35	0.016
Hemoglobin (g/dL)	12.1	12	11	1.32	0.27
Platelets (X 10^3/μL)	157.1	211.3	176.9	3.36	0.039
Spleen weight (gm)	649.1	532.3	821.3	0.37	0.69
Spleen volume (cm^3)	1431	1026.8	1908.2	1.06	0.35

ANOVA, analysis of variance; WBC, white blood cells

© 2021 Erez L et al. *JAMA Network Open.*

eTable 3. Baseline Characteristics of Trauma Patients Who Underwent Splenectomy

Clinical Parameter, No. (%)	N=24
Age in y, median (range)	35 (19-90)
Gender	
Male	18 (75)
Female	6 (25)
Medical background	
ITP	1 (4)
Autoimmune disorder	1 (4)
Alive at the time of analysis	22 (92)
Baseline laboratory parameters, median (range)	
Hemoglobin (g/dL)	12 (6.7-14.7)
WBC (X 10^3/μL)	15 (7.9-33.8)
Platelets (X 10^3/μL)	210 (83-406)
Prior malignancy	0
Clinical splenomegaly	0
Pre-surgery imaging results	
Not relevant	24 (100)

WBC, white blood cells; ITP, immune thrombocytopenic purpura

© 2021 Erez L et al. *JAMA Network Open.*

eTable 4. Surgical-Pathology Data for Trauma Patients Who Underwent Splenectomy

Clinical Parameter, No. (%)	N=24
Surgery type	
Laparoscopy	1 (4)
Laparotomy	23 (96)
Duration of hospital admission in median days (range)	15 (3-120)
Surgical complications	6 (25)
30-day mortality rate	0
Spleen weight in grams, median (range)	159 (68-464)
Spleen volume in cm^3, median (range)	323 (150-975)
Macro pathological irregularity	0
Spleen pathology resulting in new diagnosis	
No new diagnosis	24 (100)
Spleen pathology diagnosis resulted in new management decision	0

© 2021 Erez L et al. *JAMA Network Open.*
